# Viewpoint on the WebMO First 25 Years Symposium at the Fall 2025 ACS Meeting

**DOI:** 10.1021/acs.jpca.6c03169

**Published:** 2026-07-27

**Authors:** Carl Salter, James B. Foresman, J. R. Schmidt, William F. Polik

**Affiliations:** † Department of Chemistry, 1688 Moravian University, Bethlehem, Pennsylvania, 18018, United States; ‡ Kinsley School of Engineering, Sciences and Technology, 8752 York College of Pennsylvania, York, Pennsylvania, 17403, United States; § Theoretical Chemistry Institute and Department of Chemistry, University of Wisconsin−Madison, Madison, Wisconsin, 53706, United States; ∥ Department of Chemistry, 3930 Hope College, Holland, Michigan, 49423, United States

## Abstract

Computation has become an essential tool for understanding and predicting chemical phenomena. However significant access, cost, and technical barriers limited its widespread use in education and research. WebMO is a web-based interface to most popular computational chemistry programs that allows chemistry students, faculty, and researchers to perform calculations and visualize the results using only the web browser on their computer. This lowering of technical and cost barriers has extended access to state-of-the-art computational chemistry throughout educational and research communities. An ACS Symposium invited a broad range of speakers to share their experiences using WebMO to teach chemical concepts, explain experimental results, and enable research projects. The use of WebMO as platform to collaborate in developing and sharing educational and research experiences was discussed. Interconnectivity with other software tools and future directions of WebMO were also explored. This Viewpoint article summarizes the perspectives of 19 speakers on how WebMO has influenced the delivery of computational chemistry within the chemistry community over the past 25 years and offers ideas for future directions.

## INTRODUCTION

The American Chemical Society (ACS) Division on Computers in Chemistry (COMP) held a day-long symposium entitled “WebMO: The First 25 Years” on August 15 at the Fall 2025 ACS meeting in Washington, DC.^1,2^ The symposium was organized by Carl Salter and Jim Foresman and included 19 speakers from high schools, colleges, universities, institutes, chemistry consortia, and software development projects.

WebMO is a web-based interface to computational chemistry programs.^3^ It was initially developed in 2000 for undergraduate research students as an alternative to using the command line and a text editor for creating, running, and retrieving quantum chemistry calculations. Molecules could be created with an integrated 3-D editor, jobs submitted to several popular programs, and output files were parsed and displayed in tables. In 2003, a Java applet was added for visualizing molecular orbitals. Subsequently, support for additional computational programs was added, management features for user accounts were introduced, and integration with external queuing systems was implemented. In 2017, Java was replaced with modern HTML5/Javascript technology, resulting in a fully browser-based experience with no software installation by the user. This also allowed for more powerful features such as interactive tables and spectra. Most recently, a Python interface has been added that allows for automation of job submission and result retrieval, as well as subsequent analysis of computational results.

The stated mission of WebMO is that “WebMO will increase access and lower barriers to using state-of-the-art computational chemistry in teaching and research”. WebMO accomplishes this by allowing chemistry students and researchers to run a wide variety of computational chemistry calculations from the web browser on their computer. WebMO provides reasonable defaults and visual representations of results for novices. It also provides full access to input and output files for experts. WebMO lowers technical and cost barriers by eliminating the need to install any software by users on their computers and by sharing a single installation of programs on a server among all users.

The broad goal of the symposium was to share the use and impact of WebMO software over the last quarter century with the broader chemistry community. Many examples were given of using WebMO to teach chemistry and promote research. Several consortia using WebMO were described. Complementary interfaces for using computational chemistry were presented, along with opportunities for collaboration with WebMO. Future directions for WebMO were explored.

Various broad themes emerged from the presentations, including:

- Importance of computation in science and education
- Challenges with using computational chemistry programs
- Promoting computational chemistry in the undergraduate curriculum
- Enabling research projects by undergraduate students and nonspecialists
- Software tools for consortia to support teaching and research collaborations
- Connectivity and collaboration among chemistry software packages
- Philosophy behind WebMO development and future directions

## SYMPOSIUM REPORT

While many speakers touched on several of the broad themes, this report is organized by the primary area on which each presented.

### Use in Chemistry Education

Many presentations described how WebMO was useful in enhancing student comprehension of fundamental chemical concepts and also in illustrating the importance of computation in understanding and predicting chemical behavior.

**Carl Salter of Moravian University** provided an introduction on how WebMO is a widely accessible platform for teaching and visualizing computational chemistry in the undergraduate curriculum. Salter pointed out that it enables educators to share exercises they developed, for example, activities developed by MoleCVUE consortium members.^4^ Salter described an activity he developed that explores the isomers of C_4_H_4_.^5,6^ Students list possible isomers, predict their relative stabilities, and validate their predictions using ab initio calculations. The computational results are then used to determine total bond energies and heats of formation of the isomers.

**Kevin Range of Commonwealth University at Lock Haven** described how WebMO and Gaussian^7^ are used to enhance organic chemistry pedagogy. The WebMO builder allows students to quickly explore constitutional and stereoisomers, and the built-in mechanics calculation provides a qualitative ordering of their relative energies. Electronic structure calculations using a program like Gaussian allowed students to make conformational energy diagrams with WebMO’s coordinate scan functionality, as well as calculate IR and NMR spectra and relate the peaks to molecular structure using WebMO’s interactivity between tables and spectra. The relative acidity and basicity of protons is investigated by computation and compared to CARDiO and ARIO predictions.^8,9^ Overall, the combination of WebMO and Gaussian is a useful general-purpose tool for understanding organic chemistry spectroscopy and predicting reactivity.

**Angela Migues of SUNY Oneonta** demonstrated how WebMO was used to explore Brønsted–Lowry Acid Base theory through protonation at nitrogen versus oxygen atoms. In the first example, students calculate energetics of protonation of water versus ammonia and determine that the protonation at nitrogen is more favored than at oxygen. In the next set of examples, students investigate several alcohol–amines, such as methanolamine, ethanolamine, etc. Upon computing the reaction energy for protonation at the amine versus the alcohol group, they find that protonation at the amine is energetically more favorable. Students then predict where protonation will occur in more complex molecules, specifically tris­(hydroxymethyl)­aminomethane (commonly referred to as “tris”) and diethylenimide oxide (commonly known as morpholine). The goal of this activity is for students to apply their computational observations toward gaining insight into chemical reactivity.

**James Foresman of York College of PA and Gaussian, Inc.** described the use of WebMO with Gaussian for a wide variety of learning activities, including structural and vibrational analysis of C_4_H_4_ isomers, ^13^C FT-NMR studies of nitro-toluidines, absolute configuration of a flexible chiral compound using conformational analysis, comparing basic and acidic forms of phenolphthalein, finding transition structures of metal hydrides to form metal oxides, and protein–ligand interactions using the ONIOM model.

**George Shields of Furman University** described how WebMO has been used to introduce physical chemistry concepts into their chemistry curriculum.^10^ General chemistry computational laboratories include Valence Shell Electron Pair Repulsion Theory (VSEPR) and the shape of molecules, solving the Schrödinger equation for diatomic molecules, and using a high-level model chemistry to calculate Δ*E*, Δ*H*, and Δ*G* for the combustion of H_2_ and CH_4_. Physical chemistry laboratories use HF theory and the cc-pVNZ basis sets to illustrate convergence to the HF limit for H_2_^+^, and MP2 and CCSD­(T) to investigate correlation energy. Students learn how DFT models, MP2, CCSD, and CCSD­(T) vary for computing the dissociation of H_2_ as a function of theory and basis set. WebMO is used to calculate the MOs and match them with the experimental photoelectron spectrum for H_2_O, CH_4_, and C_2_H_4_.

**Robert Gotwals of the North Carolina School of Science and Mathematics** described his computational chemistry curriculum for high school students at NCSSM.^11^ He uses Gaussian,^7^ MOPAC,^12^ and WebMO to teach approximately 30 high school students each year in the course “Introduction to Computational Chemistry”. In addition, NCSSM supports the statewide North Carolina High School Computational Chemistry server that is available to any precollege student or teacher physically residing in the state. Gotwals and Shawn Sendlinger (NC Central University) have developed several dozen exercises, which are online at the Shodor/CCCE Web site^13^ and are described in their forthcoming textbook *Beginner’s Guide to Computational Chemistry*.^14^ Gotwals and Sendlinger also ran workshops at Shodor and CCCE for faculty to incorporate computational chemistry into their teaching.

**Joseph Baker of The College of New Jersey** reported that TCNJ uses WebMO as the foundational platform for integrating computational chemistry across multiple levels of the curriculum and into authentic undergraduate research. In General Chemistry, students use WebMO to visualize molecules and explore their 3D geometries, providing a practical computational aspect to their study of VSEPR theory. Physical chemistry students use WebMO as an interface to Gaussian for calculating the IR and UV–Vis spectra of molecules such as atmospheric gases and conjugated dyes which allows for direct comparison between theoretical predictions and experimental results. In a computational chemistry Course-based Undergraduate Research Experience (CURE),^15^ students use WebMO with Gaussian to calculate the electrostatic potentials of small molecules and protein-bound ligands, which is an important step in parametrizing the molecules for subsequent molecular dynamics simulations using Amber software.^16^

**Matthew Sonntag of Albright College** described the use of WebMO for laboratory exercises that cross traditional disciplinary boundaries. One exercise involved examining electrophilic aromatic substitution by using NMR, molecular orbital, and partial charge calculations to predict the location of substitution.^17^ Another exercise combined Raman, IR, and computational tools to gain an understanding of selection rules in two different hexyne molecules.^18^ A third project involved the synthesis and spectroscopic properties of triphenylphosphine chalcogens.

**Matthew Zwier of Drake University** described usage of WebMO to support guided inquiry learning in first-semester general chemistry. Students use WebMO to discover the shapes and energy ordering of atomic orbitals and the energetics of how orbitals fill. For instance, students discover that triplet atomic oxygen (electrons spread out in p orbitals) is substantially lower in energy than singlet oxygen (electrons maximally paired). They further work out the Aufbau filling of orbitals, one electron at time from Sc­(III) to Sc(0), and observe the interplay between electron repulsion and nuclear attraction that gives rise to d-block electron configurations. WebMO is also used in inorganic, physical, and computational chemistry courses at Drake.

These presentations demonstrated the use of WebMO across the chemistry curriculum to develop and enhance understanding of chemical concepts. The widely varying examples illustrate the breadth of computational chemistry and versatility of the WebMO interface.

### Use in Chemistry Research

WebMO was a described as a useful tool that allows undergraduate students to use computational chemistry to conduct and publish peer-reviewed research projects.

**Keith Kuwata of Macalester College** described how the WebMO interface has made it easier for undergraduates to make significant contributions to atmospheric chemistry research projects. Juniors and seniors taking his computational chemistry class have earned coauthorships on papers based solely on their work during the semester. For example, students used WebMO in calculating the kinetics of vinyl hydroperoxide formation, isomerization, and decomposition under atmospheric conditions and then contributed to a 2018 *Journal of Physical Chemistry A* paper.^19^ WebMO also facilitated computational chemistry research by enabling students to easily apply multiple electronic structure codes to their molecules. For example, in an ongoing study of the reaction of acetonylperoxy and hydroperoxy radicals, students have used Gaussian^7^ to obtain optimized geometries and vibrational frequencies, Orca^20^ to obtain DLPNO-CCSD­(T) electronic energies, and Q-Chem^21^ to calculate singlet–triplet energy gaps. WebMO also forms the common user interface to computational chemistry resources shared by the Midwest Undergraduate Computational Chemistry Consortium (MU3C), which has enhanced student research productivity and enabled student participation at regional and national ACS conferences.^22,23^

**Dani Kohen of Carleton College** described how WebMO enabled her to use quantum chemistry in her research and teaching. While trained as a theoretician and previously doing molecular dynamics simulations in condensed phases, Kohen was asked to teach a computational chemistry class, which was enabled by using WebMO. In addition, she and her course and research students computed the effects of sterics and electronics on silicon-metal bonding, which resulted in three publications.^24–26^ Overall, WebMO allowed students to carry out course-related and new research using quantum chemistry and then publish their work.

### Use to Support Consortia Activities

While the following presentations describe educational and research uses of WebMO, they also illustrate how WebMO is being used as a common interface by communities of educators and researchers for sharing their resources and experiences with each other.

**Joanne Stewart of Hope College** described how WebMO enabled inorganic chemistry instructors to bring computational chemistry into the classroom by lowering the barrier to drawing, calculating, and visualizing results. Stewart described examples of student-centered computational exercises on bonding, symmetry, molecular orbital theory, and predicting spectroscopic results from the Interactive Online Network of Inorganic Chemists (IONiC) on their Virtual Inorganic Pedagogical Electronic Resource (VIPEr) website.^27^ These exercises enable students to visualize, predict, discover, and connect laboratory experiments with theory.

**Heidi Hendrickson of Lafayette College** reported how WebMO can be used in inorganic chemistry courses to enable students to interact with point group symmetry elements in three dimensions. Students could predict and test how changes in molecular structure impact point group symmetries using WebMO. Hendrickson described use of an activity developed by Dr. Alex Davis of Cabrini University and distributed through the MoleCVUE Consortium.^28^ The first part of the activity teaches students to build a molecule using the WebMO interface, optimize the structure, examine the symmetry elements, and determine the point group. The second part of the activity reverses the task, asking students to build molecules that have particular point group symmetries, enabling them to carefully consider how changes in the structure of a molecule can impact its symmetry. The interactivity of WebMO allowed students to go through both directions of this exercise, and thereby gain a deeper understanding of symmetry elements and point groups.

**Ashley Ringer McDonald of Cal Poly San Luis Obispo** reported that WebMO launched a new era in chemistry education in which computational chemistry using research-quality software could be effectively implemented in the undergraduate chemistry curriculum. This has resulted in synergy with other educational groups such as Psi4Education^29^ and Molecular Sciences Software Institute (MolSSI),^30^ who are leveraging WebMO to teach computational chemistry to students. Currently, there is a significant effort to teach students in data-driven approaches in chemistry, which requires skills like programming, data analysis, data visualization, and machine learning. Resources from MolSSI were described that offer students and faculty a wide range of help to develop these kinds of cyberinfrastructure skills and incorporate them into the chemistry curriculum.

These and earlier presentations demonstrate how WebMO is being used as a common interface by consortia of chemists such as IONiC/VIPEr, MoleCVUE, Psi4Education, MolSSI, and MU3C.

### Interfacing Computation with Other Software

Features of WebMO that allow interaction with other software packages were described, along with opportunities for interconnectivity and collaboration.

**J. R. Schmidt of the University of Wisconsin** and a codeveloper of WebMO described several ways to extend the utility of WebMO, using its built-in capabilities. WebMO can import structures by name or from databases, and it exports structures in a wide variety of formats. WebMO jobs are defined through calculation templates, and new customizable job types can be defined through a built-in template editor and variable creation system.^31^ Groups of users (students) and their administrators (instructors) provide separation of WebMO usage by course or research group, as well as the transfer of administrative responsibilities from the system and WebMO administrators to instructors.^32^ Finally, the recent Python interface to WebMO allows for programmatic control of job submission, the retrieval of results and images, and the ability to perform custom analyses on computed results.^33^ This allows for automated job submission and customized viewing of job results. Taken as a whole, these features allow users to customize and extend WebMO for their own purposes.

**Jason Sonnenberg of Wolfram Alpha** reported that WA is also working to reduce the barriers to using computational chemistry and suggested that coding provides a useful way to teach chemistry to a wider audience. The WA chemistry team recently introduced quantum chemistry tools that allow one to easily compute and display normal modes and molecular orbitals in WA’s digital notebook environment.^34^ Two forward-thinking ideas were to use the PySCF package to implement their calculations and to interface with WebMO’s REST interface for submitting and importing computational results.

**Geoff Hutchison of the University of Pittsburgh** described the use of several tools that extend the usage of WebMO. Avogadro allows the building of not only molecules, but also more-complex systems, such as clusters, nanotubes, and polymers, which can then be submitted for computation through WebMO.^35^ Open Babel can generate conformers of a structure for subsequent calculation.^36^ Opportunities for further integration between Avodagro and WebMO were suggested, such as drop-and-drag of structures between programs and direct submission of jobs from Avogadro to WebMO, which should now be possible through WebMO’s new REST interface. Hutchison emphasized the importance of having general-use tools like WebMO and Avogadro that offer integration across multiple packages, as various programs offer different calculations and capabilities. Additionally, while many developers focus on new computing techniques, ease-of-use is also critical to allow practicing scientists to focus on the using these methods for their applications.

**Daniel Crawford of Virginia Tech** provided an overview of how the Molecular Sciences Software Institute (MolSSI) supports computational molecular science.^37^ MolSSI involves a broad community of quantum, biomolecular and macromolecular simulation, and materials scientists. MolSSI provides open-source software for computational scientists, including the Basis Set Exchange (BSE),^38^ QCArchive,^39^ the MolSSI Driver Interface (MDI),^40^ and the Simulation Environment for Atomistic and Molecular Modeling (SEAMM).^41^ MolSSI’s Education Initiative^42^ provides open-source educational resources through in-person workshops and online courses on computational science infrastructure such as Python, C++, git, and applications in quantum chemistry and simulation methods.

These presentations described how WebMO interacts with other ongoing software projects to support the use of computational chemistry in education and research.

### Guiding Principles and Future Directions

The concluding presentation provided the historical context of the development of WebMO project, as well as an outlook on its future directions.

**Will Polik of Hope College** and a codeveloper of WebMO provided a perspective of how WebMO began by utilizing the World Wide Web and incorporating various technologies (Java applets, JavaScript, HTML5) to enable new capabilities (MO rendering, interactive plots). Several guiding principles have been consistently followed over the past 25 years in the development of WebMO,^43^ including:

- Engine agnostic – equal support for all popular programs
- No end-user software installation – only a web browser is needed
- Support multiple skill levels – reasonable defaults for novices, and full access to input and output files for advanced users
- User-friendly visualization of output – tables, plots, images, and animations
- Useful across the entire chemistry curriculum – one tool for many courses
- Rapid incorporation of new computing paradigms – Java, JavaScript, HTML5
- Connectivity to other computing standards – import, export, spreadsheets, REST, and Python
- Active support of the computational chemistry community – free email support, workshops, conference participation

Future directions for WebMO were also described, including the Python interface for job submission and result retrieval, support of molecular dynamics programs, and providing WebMO as a cloud-based service to further increase access.

## CONCLUSIONS

Computation is a tool that aids in the understanding of chemical concepts in all chemistry courses and in modeling and predicting chemical phenomena in chemistry research. While the technical challenges to implement, run, and interpret computational chemistry can be significant, an interface like WebMO greatly lowers cost and usability barriers. However, it is the innovative work by chemistry faculty to develop educational exercises and use it in research that ultimately puts WebMO and computational chemistry in the hands of students. In addition to direct educational use, WebMO also has had impact in supporting the work of communities of educators and researchers by providing a common interface that allows sharing of effort, experience, and resources. Finally, the guiding principles behind WebMO serve as a model for how a software product can support a broad range of users and multiple purposes. The WebMO developers are extremely appreciative of the efforts and accomplishments of the presenters and thank them for offering their perspectives and experience.
